# Characterization of microflora in Latin-style cheeses by next-generation sequencing technology

**DOI:** 10.1186/1471-2180-12-254

**Published:** 2012-11-07

**Authors:** Tina S Lusk, Andrea R Ottesen, James R White, Marc W Allard, Eric W Brown, Julie A Kase

**Affiliations:** 1 Oak Ridge Institute for Science and Education, Oak Ridge, TN 38730, Tennessee; 2 Division of Microbiology, Center for Food Safety and Applied Nutrition, US Food and Drug Administration, 5100 Paint Branch Parkway, College Park, MD 20740, MD, USA; 3 Institute for Genome Sciences, University of Maryland School of Medicine, 801 W. Baltimore St., Baltimore, MD 21201, MD, USA

**Keywords:** Latin-style cheese, Next Generation Sequencing, Microflora, Bacteria, *Exiguobacterium*

## Abstract

**Background:**

Cheese contamination can occur at numerous stages in the manufacturing process including the use of improperly pasteurized or raw milk. Of concern is the potential contamination by *Listeria monocytogenes* and other pathogenic bacteria that find the high moisture levels and moderate pH of popular Latin-style cheeses like queso fresco a hospitable environment. In the investigation of a foodborne outbreak, samples typically undergo enrichment in broth for 24 hours followed by selective agar plating to isolate bacterial colonies for confirmatory testing. The broth enrichment step may also enable background microflora to proliferate, which can confound subsequent analysis if not inhibited by effective broth or agar additives. We used 16S rRNA gene sequencing to provide a preliminary survey of bacterial species associated with three brands of Latin-style cheeses after 24-hour broth enrichment.

**Results:**

Brand A showed a greater diversity than the other two cheese brands (Brands B and C) at nearly every taxonomic level except phylum. Brand B showed the least diversity and was dominated by a single bacterial taxon, *Exiguobacterium*, not previously reported in cheese. This genus was also found in Brand C, although *Lactococcus* was prominent, an expected finding since this bacteria belongs to the group of lactic acid bacteria (LAB) commonly found in fermented foods.

**Conclusions:**

The contrasting diversity observed in Latin-style cheese was surprising, demonstrating that despite similarity of cheese type, raw materials and cheese making conditions appear to play a critical role in the microflora composition of the final product. The high bacterial diversity associated with Brand A suggests it may have been prepared with raw materials of high bacterial diversity or influenced by the ecology of the processing environment. Additionally, the presence of *Exiguobacterium* in high proportions (96%) in Brand B and, to a lesser extent, Brand C (46%), may have been influenced by the enrichment process. This study is the first to define Latin-style cheese microflora using Next-Generation Sequencing. These valuable preliminary data will direct selective tailoring of agar formulations to improve culture-based detection of pathogens in Latin-style cheese.

## Background

Latin-style cheeses continue to be highly popular in the United States, with 215 million pounds produced in 2010, up nearly 4% from 2009 [1]. Yearly per capita consumption in the United States is 0.65 pounds per person, an increase of 150% from 1997 to 2008 [2]. According to Dairy Management Inc., a non-profit group funded by dairy producers that promotes dairy products within the United States, foreign-born Hispanics constitute one-half of the US cheese consumer [3]. If migration rates remain constant and the population doubles from 2000 levels in 2050 as expected, the consumption of Latin-style cheese is likely to rise as a result [3,4].

Soft Latin-style cheeses like queso fresco typically are not aged, have a short shelf-life (about 2 weeks), and have a high moisture content (41/59%) [5]. The lack of an aging step as well as high moisture content and the moderate pH level of Latin-style cheeses can all contribute to pathogen growth and increases the likelihood of pathogens surviving and possibly multiplying to the levels necessary to cause illness [6]. For this reason, the US FDA prohibits the interstate sale of this cheese type if it is manufactured using raw milk [5]. However, for some the taste of Latin-style cheese made with raw milk is preferable.

Between 1998 and 2009, 56 cheese-associated disease outbreaks occurred in the United States resulting in 1,377 illnesses, 171 hospitalizations, and 2 deaths [7-9]. Eighteen of these occurrences (32%) specifically involved Latin-style cheeses and a variety of pathogens, resulting in 212 illnesses (15% of total), 95 hospitalizations (55%), 2 deaths (100%), and at least 7 stillbirths [10]. Individuals making homemade cheese (i.e. bathtub cheese) sold in grocery stores accounted for 85 illnesses [7-9,11]. The most serious outbreak involving Latin-style cheeses occurred in 1985; 142 cases of listeriosis caused 48 deaths, of which 30 involved neonates or fetuses [10].

In response to a foodborne outbreak, suspect samples are analyzed according to standardized methods including those described in the FDA Bacteriological Analytical Manual (BAM). One goal of analysis is to recover isolated colonies of the pathogenic bacteria that can assist in matching any recovered clinical, food, and environmental isolates to determine the source(s) of illness. Most methods described in the FDA BAM begin with enriching the suspected food product in a universal or microbe-specific enrichment broth for up to 24 hours. The sample is then plated onto selective agar specific for the target bacteria to obtain isolated colonies. The initial enrichment step is designed to recover and propagate bacterial pathogens in the product facilitating downstream detection efforts. However, enrichment can also influence levels of background microflora. A food sample may consist of a complex consortium of bacteria that can out-compete and otherwise hinder efforts to recover human pathogens. With improved characterization of the microbial taxonomy and abundance associated with a given enriched food product, broths and agar formulations can be vastly improved in terms of culture selectivity.

Several studies have attempted to describe the full range of microbes present in cheeses as well as in various steps along the manufacturing and maturation process to understand temporal microflora changes [12-18]. The most widely-used approach begins with the plating of cheese samples on agar and picking isolated colonies for subsequent identification using biochemical analyses or molecular characterization. These methods are labor intensive and inherently biased [19,20]. For this reason, culture-independent techniques, including single stranded confirmation polymorphisms (SSCP) analysis of DNA and restriction fragment length polymorphism (RFLP) typing of isolates, have been used increasingly to study the bacterial populations in milk and/or cheese [20]. Next Generation Sequencing (NGS) techniques are extremely useful because of the enhanced sequencing depth that can be achieved compared to previous technologies for relatively low cost without the bias introduced by culture techniques. To date, NGS methods have been applied most prolifically to describe the human microbiome [21], but they have also been widely used to describe a vast array of environmental and agricultural ecologies, including microflora of trees [22] and tomato surfaces [23], and even for epidemiological approaches in hospital pathogen tracking [24]. This technology has also been used to study the bacterial diversity of other cheeses as well, including artisanal cheeses [25], traditional Polish cheeses [26], and Danish semi-hard cheese [27]. However, the application of NGS methods to evaluate food microbiomes is still in its infancy.

## Results

We recovered 3708 high-quality 16SrRNA gene sequences with an average sequence length of 370bp and 309 ± 92.6 (SD) sequences per enriched cheese sample. From the four replicate Brand C cheese samples, a total of 1284 ± 92.8 sequences were recovered, 1187 ± 137.55 sequences were recovered from Brand A cheese, and Brand B produced 1237 ± 59.1 sequences. To compare environments for differentially-abundant taxonomic groups at the 0.05 significance level, Metastats (a program designed to identify significant taxonomic differences between microbial communities) [28] was used for phylum, class, order, family and genus level assignments. Average abundance of bacterial classifications are presented in Table 1 along with p-values of brand comparisons.

**Table 1:** Average abundance (%) of sequences assigned to taxa in all cheese brands

	**Classification**	**Brand A (%)**	**Brand B (%)**	**Brand C (%)**	**Significant Difference? (p ≤ 0.05)**
**Phylum**	Firmicutes	68	100	81	(A and B, p = 0.006);
					A and C, p = 0.135;
					B and C, p = 0.0)
	Proteobacteria	29	0	19	(A and C, p = 0.141;
					A and B, p = 0.0;
					B and C, p = 0.012)
**Class**	*Clostridia*	66	0	0	(A and C, p = 0.004;
					A and B, p = 0.01)
	*Gammaproteobacteria*	22	0	19	(A and C, p = 0.65;
					A and B, p = 0.005;
					B and C, p =0.0)
	*Bacilli*	2	100	81	(A and B, p = 0.0;
					A and C, p = 0.0;
					B and C, p = 0.011)
**Order**	*Clostridiales*	67	0	0	(A and C, p = 0.003;
					A and B, p = 0.004)
	*Lactobacillales*	0	0	22	(A and C, p = 0.005;
					C and B, p = 0.006)
	*Enterobacteriales*	9	0	14	(A and C, p = 0.03;
					A and B, p = 0.002;
					B and C, p = 0.012)
	*Pseudomonadales*	9	0	5	(A and C, p = 0.049;
					A and B, p = 0.049
					B and C, p = 0.017)
	*Bacillales*	2	100	59	(A and B, p = 0.0;
					A and C, p = 0.0;
					B and C, p = 0.0)
**Family**	*Incertae Sedis XII*	0	96	45	(A and B, p = 0.0;
					A and C, p = 0.0;
					B and C, p = 0.0)
	*Staphylococcaceae*	0	3	0	(A and B, p = 0.01;
					B and C, p = 0.01)
	*Planococcaceae*	0	0	14	(A and C, p = 0.002;
					B and C, p = 0.004)
	*Streptococcaceae*	0	0	22	(A and C, p = 0.005;
					B and C, p = 0.007)
	*Clostridiaceae*	67	0	0	(A and B, p = 0.007;
					A and C, p = 0.004)
	*Enterobacteriaceae*	9	0	14	(A and B, p = 0.002;
					A and C, p = 0.025;
					B and C, p = 0.01)
	*Pseudomonadaceae*	7	0	5	(A and B, p = 0.008;
					A and C, p = 0.12;
					B and C, p = 0.04)
**Genus**	*Exiguobacterium*	0	96	45	(A and B, p = 0.0;
					A and C, p = 0.0;
					B and C, p = 0.0)
	*Kurthia*	0	0	14	(A and C, p = 0.001;
					B and C, p = 0.003)
	*Clostridiaceae*	68	0	0	(A and B, p = 0.006;
					A and C, p = 0.002)
	*Raoultella*	7	0	10	(A and B, p = 0.002;
					A and C, p = 0.18;
					B and C, p = 0.012)
	*Pseudomonas*	7	0	5	(A and B, p = 0.008;
					A and C, p = 0.16;
					B and C, p = 0.034)
	*Lactococcus*	2	0	22	(A and B, p = 0.006;
					A and C, p = 0.004;
					B and C, p = 0.006)
	*Staphylococcus*	0	3	0	(A and B, p = 0.01;
					B and C, p = 0.009)
	*Enterobacteriaceae_Other*	0	0	2	(A and C, p = 0.008;
					B and C, p = 0.018)

Taxa represented occurred at ≥ 1% abundance of the total for each brand.

### Taxonomic distributions among samples

After assigning sequences to a taxonomic lineage using the RDP Bayesian classifier, we first examined the phylum level distributions across all enriched cheese samples and found fairly similar 16S rRNA profiles between all three cheese brands (Table 1). Firmicutes dominated the observed sequences in all cheese samples, with the highest proportions found in all four Brand B samples (100%), the next highest in Brand C (71-88%), and the lowest in Brand A (56-82%). Brand A and Brand C samples were more diverse at the phylum level than Brand B, with Proteobacteria constituting 12-29% of sequences from Brand C samples and 18-43% of Brand A samples.

Differences between the cheeses become more evident at class level classification. Brand A samples have a significantly different profile than the other two cheese brands. Class-level abundance profiles for Brand C and Brand B samples are clearly dominated by *Bacilli* taxa, while Brand A appears to be dominated by *Clostridia* (49-82%). *Gammaproteobacteria* comprise the majority of the remaining diversity for Brands A and C with 17-26%, and 12-29%, respectively.

Similarities are shared by Brand B and Brand C at the genus level (Table 1). Both are dominated by *Exiguobacterium*, though it constitutes nearly all Brand B abundance at 96% while it shows lower abundance in Brand C at 45%. Not surprisingly, Brand C shows much more diversity than Brand B at the genus level, with 6 operational taxonomic units (OTU) compared to only 2 identified in Brand B. Unlike the other brands, Brand A is dominated by *Clostridiaceae* (68%) at the genus level. Brands A and C share 3 OTUs – *Raoultella*, *Pseudomonas*, and *Lactococcus*. However, only *Lactococcus* was significantly more abundant (p-value = 0.004) between the brands, with Brand C consisting of 22% of this classification versus 2% of Brand B.

Within each cheese brand, abundance percentages for dominant OTUs in the genus classification are similar to those at the order and family classifications from which they descended. For instance, Brand B is comprised of the family *Incertae Sedis XII* (96%) within the order *Bacillales* (100%), which is not surprising since this brand is almost entirely dominated by a single classification (*Exiguobacterium*) at the genus level that falls within the family *Incertae Sedis XII*. Similar to Brand B, Brand C is also dominated by *Incertae Sedix XII* (45%) and Bacillales (59%), as well as *Exiguobacterium* (46%) at the genus level. Brand A is dominated by *Clostridiaceae* (67%) at the family level, which falls within the order *Clostridiales* noted in Brand A at 67% abundance. *Clostridiaceae* dominates Brand A at the genus level with 68%, which falls within the *Clostridiaceae* family.

**Figure 1:**
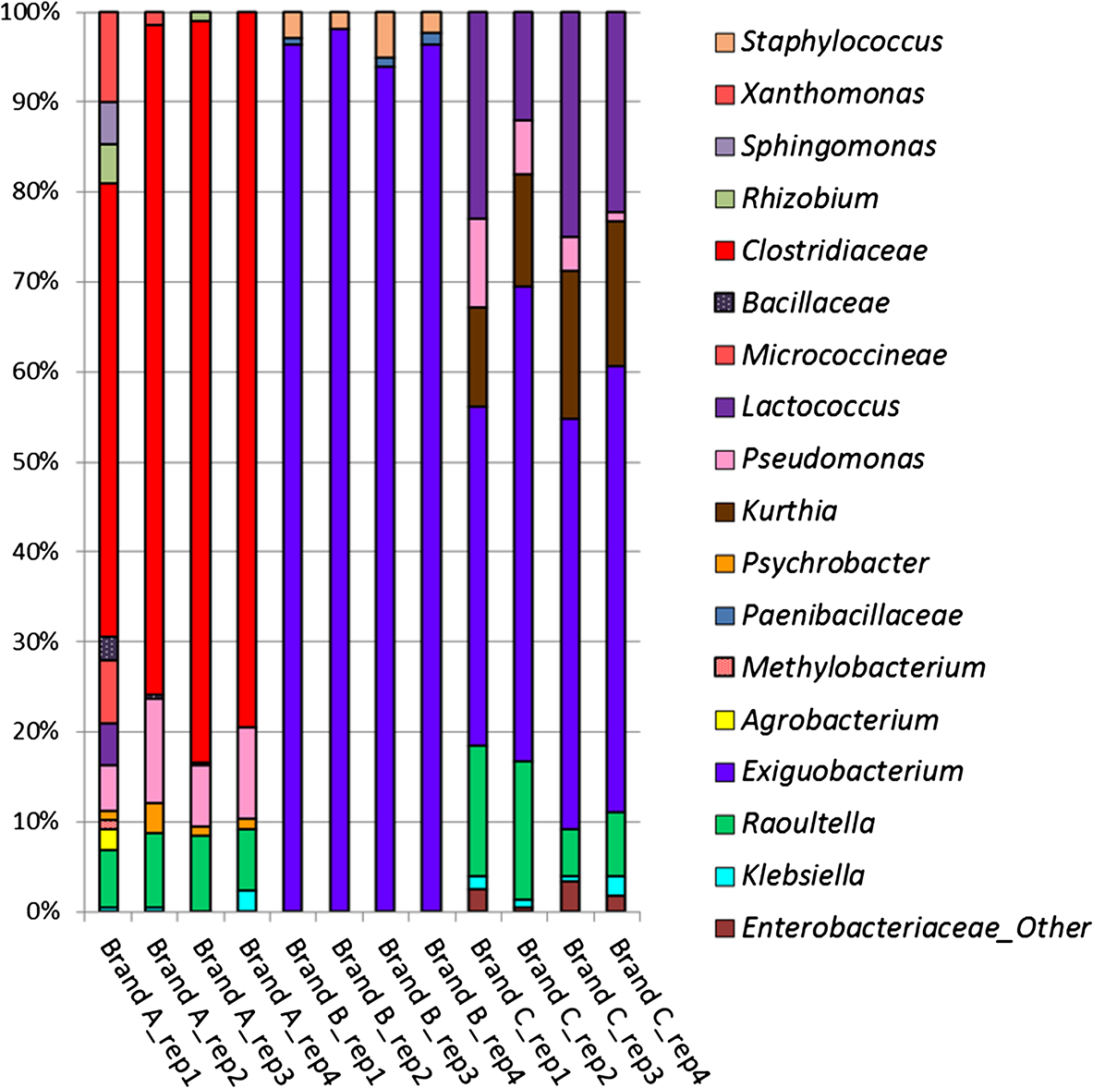
**Genus level abundance profiles using 16S rRNA sequence classifications.** Taxa represented occurred at ≥ 1% abundance in that sample.

**Figure 2:**
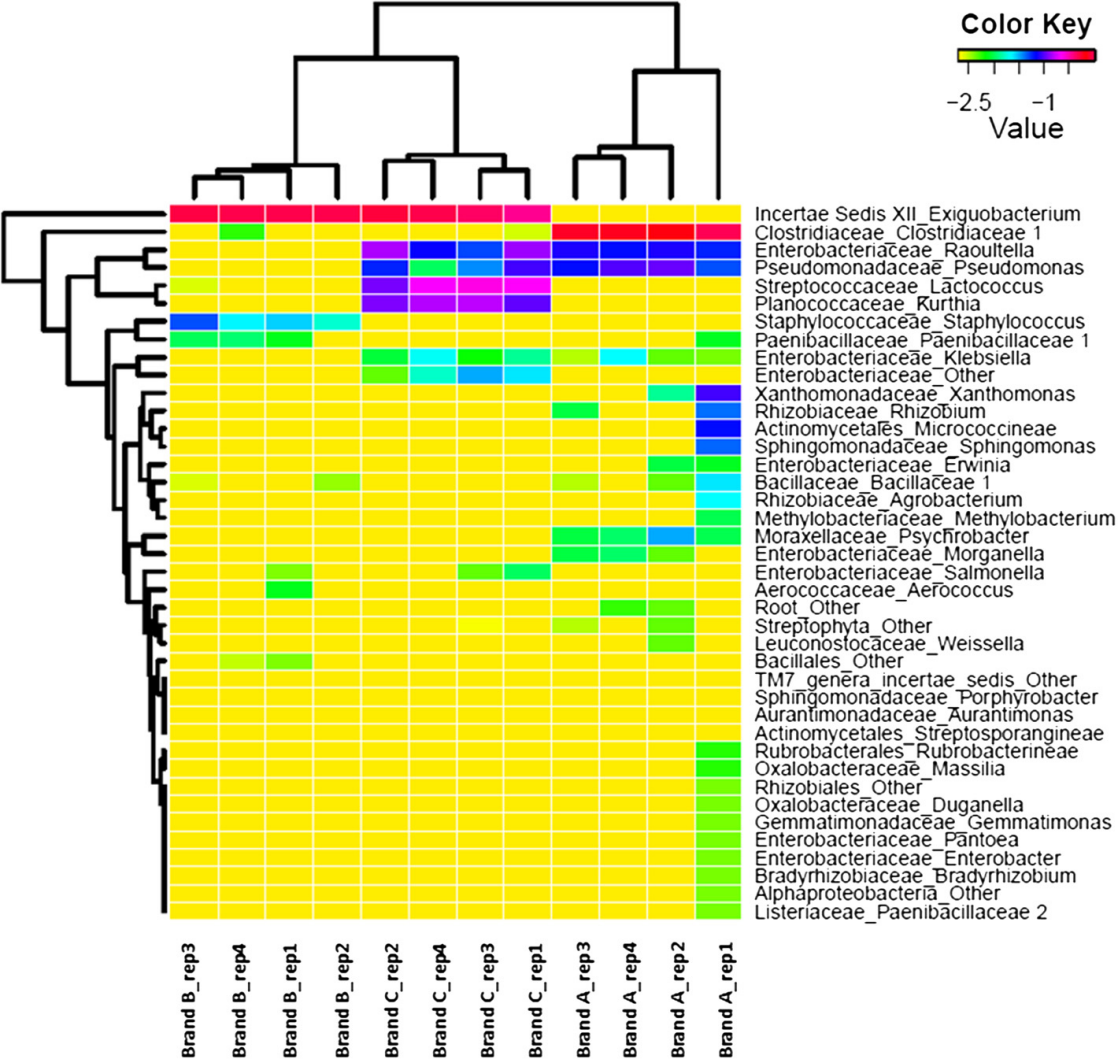
**Hierarchical clustering of samples using Genus level distributions.** Displayed values are log transformed relative abundances within each sample, (e.g. 0.10 ~ −1; 0.01 ~ −2). Visualized using skiff in CloVR.

The diversity and uniqueness of Brand A cheese is partially explained by a replicate within Brand A, replicate Brand A_rep1, that appears to have more diversity at the class level than the other 3 replicates, with the presence of *Alphaproteobacteria*, *Actinobacteria*, and *Betaproteobacteria*, of which only *Alphaproteobacteria* is shared by Brand A_rep3 in very low abundance. This diversity is evident at the genus level as well (Figures 1 and 2), with Brand A_rep1 containing 4 operational taxonomic units (OTUs) not found in any other Brand A replicates, nor in any samples from the other cheese brands, using a 95% identity threshold for clustering sequences. In addition, Brand A_rep1 contains 13 OTUs total that occurred at a ≥ 1% abundance in the sample at the genus level, while the other Brand A replicates as well as all replicates from the other cheese brands contain no more than 7 OTUs per sample.

### Diversity analysis using operational taxonomic units

**Figure 3:**
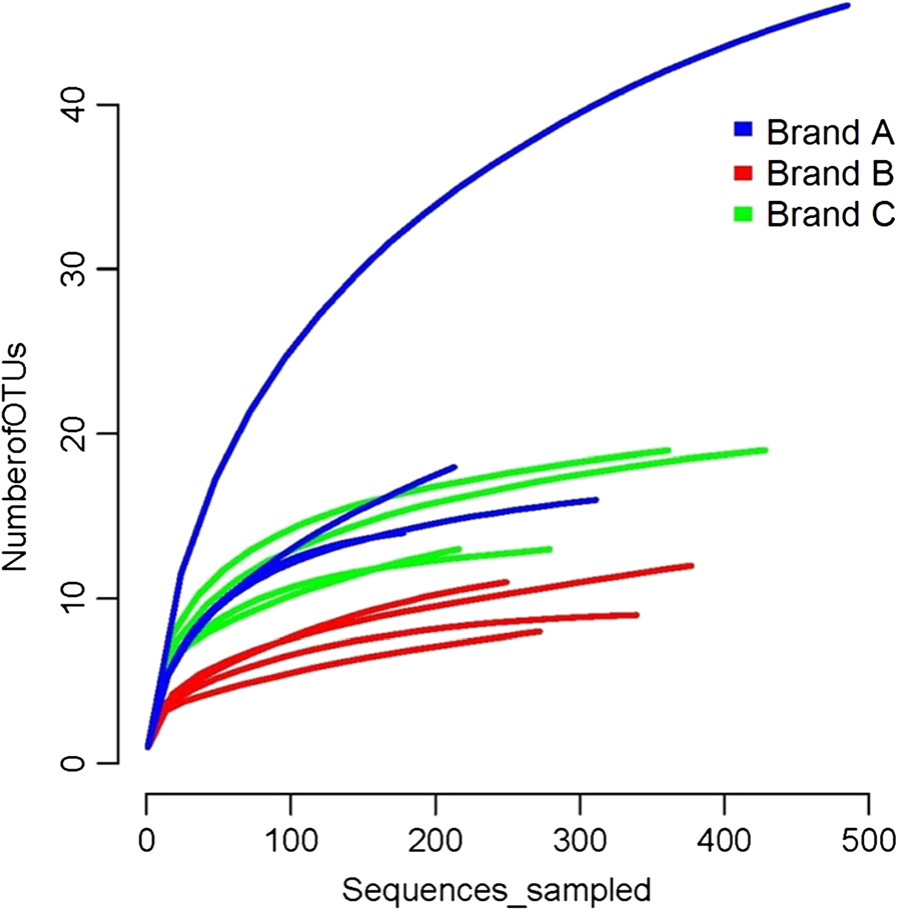
Rarefaction curves of OTUs in all 4 replicates of each cheese brand.

Rarefaction curves of all enriched cheese samples (Figure 3), also support the observation that Brand A samples supported the greatest diversity among the three cheeses. The greater diversity of Brand A cheese sample Brand A_rep1 is displayed, rising dramatically above all other samples. This is confirmed with the UniFrac metric, which shows the replicate samples of each brand distinctly clustered together by brand except for Brand A_rep1. Brand C replicates cluster together rather tightly, more so than the Brand B replicates.

### CloVR analysis

Using the automated 16S rRNA pipelines provided by the CloVR software package ( http://clovr.org). Replicates within each cheese type clustered as expected at the genus level except for the Brand A_ rep1 (Figure 2). Brand B samples show the least diversity of the cheese brands with only 2 genera identified in substantial quantities (*Exiguobacterium* and *Staphylococcus*). Brand C shows a bit more diversity, dominated clearly by *Exiguobacterium* though other genus are present including *Raoultella*, *Pseudomonas*, *Lactococcus*, *Kurthia*, and other *Enterobacteriaceae*. Brand A shares *Raoultella* and *Pseudomonas* with Brand C and low amounts of *Klebsiella*, but it is still dominated by *Clostridiaceae* with trace amounts of a variety of genera. Brand A_rep1 shows more diversity than all the other Brand A replicates, as well as, all the other cheese brand replicates.

## Discussion

This study provides the first Next-Generation Sequencing (NGS) survey of the bacterial community in Latin-style cheeses. The order *Lactobacillales* was present in significant abundance in all Brand C replicates, which is expected since lactic acid bacteria are known for their role in the production of fermented foods including cheese (Table 1). Renye et al. sampled queso fresco from Mexico, plated samples on selective agar, and subjected colonies to 16S rRNA sequencing [29]. *Lactococcus lactis*, of the order *Lactobacillales*, was found in the highest numbers in both the cheeses made with raw milk and those made with pasteurized milk. *Leuconostoc mesenteroides,* another member of the Lactobacillales order, was also abundant [29].

The genus *Exiguobacterium* of the order *Bacillales* dominated all Brand B samples in this study; however, this genus has not been previously reported in cheese [29]. Food matrices in which this genus has been identified include raw milk [30,31], however, as well as potato processing effluent and water-boiled salted duck [32,33]. *Exiguobacterium* have been identified in a wide variety of non-food matrices including surface and pond water, oral cancer tumors, hot springs in Yellowstone National Park, Siberian permafrost, coastal soil, and a saline Romanian lake [34-39]. They have also been found to be useful in bioremediation efforts [40].

Serum dextrose broth (SDB) was used in this study due to ongoing research efforts in our laboratory to enrich *Brucella* species that might be associated with this type of soft cheese. However, SDB is not particularly selective and this rich nutrient source may have allowed uncommon bacteria to out-compete other components of the original metagenomic microflora. The Jameson Effect describes the phenomenon of low abundance microbial species ceasing growth in response to a dominant population’s arrival at stationary phase [41-44]. Tran et al. explored microflora and pathogen dynamics by using selective broth and agar to isolate *Listeria* from inoculated cheese. They found that ease of isolation was not correlated with concentration of inocula, which supports the theory that microbial community composition may play a bigger role in *Listeria* inhibition than initial concentrations [43]. Due to this potential effect of broth enrichment on the sample microflora, the selective agar employed in the next step in detection is all the more crucial and must be formulated taking into account the sample microflora after enrichment.

In this study, all replicates within each cheese brand clustered well, with the exception of Brand A_rep1 in Brand A. Perhaps bacterial DNA extraction was more efficient with this sample; however, there is not a clear reason for this discrepancy since all samples were processed identically and at the same time. Insufficient homogenization is also a possibility since enriched samples were not treated to stomaching between enrichment and aliquot collection. But if this were the case, it’s curious that other samples were not similarly affected.

While the three cheese brands used in this study were similar in style, color and texture, the bacterial abundance profiles of each were very different. The cheese manufacturers were contacted for information regarding manufacturing process to elucidate possible reasons for the observed differences (Table 2). In the U.S., commercially available queso fresco is generally prepared with starter cultures; however, this may not be true for queso fresco made in other countries [5,29]. Starter cultures are used in the manufacturing process for Brands A and B cheeses (use of starter culture to manufacture Brand C cheese could not be determined), although information about the specific cultures used could not be obtained. Other information obtained from Brands A and B included pH, % moisture, salt concentration, and % fat, but substantial differences were not noted between the two brands (Table 2). Salt concentration was not available for Brand C cheese. Brand C does have the lowest pH (5.3 versus 6.2 - 6.7), however this alone may not account for the difference in microflora profiles between Brand C and the other brands. Further study would be required to discern the effect of these and similar parameters on the microflora of the cheese brands.

**Table 2:** Manufacturer-provided parameters of Brands A, B, and C cheeses

**Parameter**	**Brand A**	**Brand B**	**Brand C**
pH	6.5	6.2-6.7	5.3
% moisture	53-57%	49-52%	54.53%
Salt concentration	1.8	1.5-2.25	ND
% fat	22%	22-24.5%	21.5%
Starter used in manufacture process?	Yes	Yes	ND

ND = Not Determined.

The methods used in this study do not discern between live and dead cells because the amplification target, 16S ribosomal RNA-encoding genes, is highly conserved in bacteria regardless of viability. Efforts exist to manipulate sample preparation to detect only cells with intact membranes by sample treatment with propidium monoazide in combination with PCR amplification [45] or the generation of transcriptomes. This will improve NGS as a tool for assessing microflora of cheese at different stages of the aging process. Additionally, Renye et al. found more variety in the types of bacteria isolated from cheeses made with raw milk versus those made with pasteurized milk [29]; another public health risk best evaluated with tools that can distinguish between live and dead cells.

It is known that DNA extraction efficiency varies within and between laboratories, and that this can have an effect on subsequent microflora analysis [46]. We addressed this in a variety of ways. First, the extraction kit used to perform the DNA extractions was chosen based on data collected in which the Qiagen DNeasy Blood and Tissue kit was compared to five other commercially-available kits for the extraction of *Brucella neotomae* DNA from the same Latin-style cheeses used in this study (T. Lusk, E. Strain, and J.A. Kase, submitted for publication). The Qiagen DNeasy kit was found to produce the highest quality and quantity DNA from this matrix. All extractions were performed by a single person at one time. Lastly, four subsamples of each enriched cheese brand were extracted and sequenced, with all replicates producing similar bacterial profiles within each brand except for Brand A, in which 1 replicate showed more diversity than its counterparts.

## Conclusions

This research presents a first look at the microflora of Latin-style cheese using Next-Generation Sequencing. Our findings offer surprising insight into cheese microflora composition, with three cheese brands exhibiting unique bacterial profiles which varied in diversity and abundance of taxa. Although the cheese are visually similar (e.g. white color and soft, crumbly texture), their bacterial profiles were very different at nearly every classification level. Brand A cheese was clearly more diverse than the other two cheese brands with 13 OTUs at the genus level using a 95% identity threshold compared to 7 and 3 for Brand C and Brand B, respectively. Additionally, Brand A was dominated by different genus than Brands B and C. Brand B showed less diversity, mostly dominated at the genus level by *Exiguobacterium* which constituted 96% of its microflora composition. *Exiguobacterium* also made up 46% of Brand C’s profile, although its presence in cheese has not been previously documented though it has been found in milk. Factors such as milk, pH, starter culture, and salt concentration may have contributed to the unique bacterial composition of each cheese brand, although no particular factor was determined to be responsible for differences in abundance between the brands based on the limited available information. Overnight enrichment in a non-selective broth also may have allowed some fast-growing bacteria to out-compete and inhibit slower growing bacteria. This emphasizes the importance of examining food samples after the broth enrichment step to provide a more accurate depiction of microflora composition when trying to selectively cultivate target organisms while decreasing competing background flora. More effort is needed to fully characterize cheese microbial populations and to understand the effects of enrichment formulations on population composition. This valuable preliminary data will certainly inform future culture-based efforts.

## Methods

### Sample processing

Three cheeses of different brands were included in the study: Brand A - queso fresco Salvadoreno; Brand C - queso fresco; and Brand B - quesito Colombiano. All were Latin-style soft cheeses made with pasteurized milk and were purchased from grocery stores in the Washington, DC area.

Twenty-five gram portions of each cheese type was added to a sterile whirl-pak bag using a sterile spatula and were held overnight at 4°C, then combined with 250 mL serum dextrose broth followed by mixing via a Stomacher 400 circulator (Seward, Worthing, West Sussex, UK) for two minutes at 230rpm. The bags were then incubated at 37°C overnight. Sample volumes of 1.5 mL were then collected from each of the 3 cheese brands, four subsamples for each brand, for nucleic acid extraction using the Qiagen DNeasy Blood and Tissue Kit (Qiagen, Valencia, CA). DNA extractions were performed within 24 hours of each other by the same person. All cheeses, if not tested upon receipt, were stored at 4°C until use. All cheeses were discarded one month after purchase or by the expiration date printed on the package, if available.

### 454 sequencing

PCR amplification for the 16S rRNA bacterial gene (V1-V3) was performed using a series of forward primers and one reverse primer described in Table 3. Standard PCRs were performed using Taqman Universal PCR Master Mix (Invitrogen, Carlsbad, CA) in a 50 μL total volume (8μL genomic DNA as template, 800nM each primer, 25 μL Taqman, and 15.2 μL reagent grade water). PCRs used an initial denaturation step of 95°C for 300 seconds, followed by 29 cycles of 95°C for 60 seconds, 55°C for 60 seconds, and 72°C for 60 seconds, with a final extension of 72°C for 300 seconds. After gel-based confirmation of PCR amplification, PCR products were purified using AMPure kit (Invitrogen) to remove primers and sequences under 300 bases. Amplicons were quantified using both the Qubit fluorometer (Invitrogen/Life Technologies, Grand Island, NY) and the NanoDrop 1000 (ThermoScientific, Waltham, MA). Amplicons were analyzed on the Agilent Bioanalyzer 2100 using the High Sensitivity Lab on a Chip Reagents (Agilent, Santa Clara, CA) to ensure that smaller fragments had been removed prior to emulsion PCR preparation.

**Table 3:** Forward and reverse primers used to amplify the 16S rRNA bacterial gene of all cheese samples

MID 11	Brand A_rep1	CGT ATC GCC TCC CTC GCG CCA TCA GTG ATA CGT CTA GAG TTT GAT CCT GGC TCA G
MID 13	Brand A_rep2	CGT ATC GCC TCC CTC GCG CCA TCA GCA TAG TAG TGA GAG TTT GAT CCT GGC TCA G
MID 14	Brand A_rep3	CGT ATC GCC TCC CTC GCG CCA TCA GCG AGA GAT ACA GAG TTT GAT CCT GGC TCA G
MID 15	Brand C_rep1	CGT ATC GCC TCC CTC GCG CCA TCA GAT ACG ACG TAA GAG TTT GAT CCT GGC TCA G
MID 16	Brand C_rep2	CGT ATC GCC TCC CTC GCG CCA TCA GTC ACG TAC TAA GAG TTT GAT CCT GGC TCA G
MID 17	Brand C_rep3	CGT ATC GCC TCC CTC GCG CCA TCA GCG TCT AGT ACA GAG TTT GAT CCT GGC TCA G
MID 18	Brand C_rep4	CGT ATC GCC TCC CTC GCG CCA TCA GTC TAC GTA GCA GAG TTT GAT CCT GGC TCA G
MID 19	Brand B_rep1	CGT ATC GCC TCC CTC GCG CCA TCA GTG TAC TAC TCA GAG TTT GAT CCT GGC TCA G
MID 20	Brand B_rep2	CGT ATC GCC TCC CTC GCG CCA TCA GAC GAC TAC AGA GAG TTT GAT CCT GGC TCA G
MID 21	Brand B_rep3	CGT ATC GCC TCC CTC GCG CCA TCA GCG TAG ACT AGA GAG TTT GAT CCT GGC TCA G
MID 23	Brand B_rep4	CGT ATC GCC TCC CTC GCG CCA TCA GTA CTC TCG TGA GAG TTT GAT CCT GGC TCA G
MID 24	Brand A_rep4	CGT ATC GCC TCC CTC GCG CCA TCA GTA GAG ACG AGA GAG TTT GAT CCT GGC TCA G
REV		CTA TGC GCC TTG CCA GCC CGC TCA GTT ACC GCG GCT GCT GGC AC

### Emulsion PCR and sequencing

Amplicons were diluted to 10^7^ molecules per μL and pooled to generate a mixture containing an equimolar representation of each independent replicate for subsequent emulsion PCR. Pooled amplicons were further diluted to estimate 0.5 copies per bead to provide optimal emulsion PCR amplification. Emulsion PCR was done using the Roche Lib-A MV kit according to the manufacturer’s specifications.

Approximately 700,000 enriched beads were loaded into one-quarter region of the Roche Titanium FLX pico-titer plate for sequencing on the Titanium FLX platform according to the manufacturer’s specifications (Roche, Branford, CT).

### Initial sequence preprocessing

Raw 16S rRNA sequences and quality scores were demultiplexed using standard *sff* processing software with adapted scripts to address additional MIDS. Sequences and quality scores were then submitted to the CloVR-16S [47] pipeline for quality screening and analysis. CloVR includes a variety of widely used 16S analysis software including QIIME [48] and Mothur [49]. Only sequences ≥ 200 nucleotides in length were included in the final analysis. Sequences containing homopolymers of more than 8 bp, or average quality scores lower than 25, or ambiguous base calls were culled from the analysis. Remaining sequences were screened for chimeras using UCHIME [50] with the default parameters. The resulting chimera-free high-quality data set was analyzed by clustering sequences into operational taxonomic units at 95% identity using UCLUST, assigning taxonomy using the RDP classifier [51] (with a minimum confidence threshold of 50%) and performing additional statistical analyses with Metastats [28] and R scripts. A detailed description of the available SOP is available at ( http://clovr.org) [52].

## Authors’ contributions

JK, AO, and TL conceived the study and participated in its design. AO and TL performed all lab work. JW performed data analysis. TL drafted the manuscript. AO, JW, JK, MA, and EB contributed to the draft of the manuscript. All authors read and approved the final manuscript.
